# Effects of cyclic changes in population size on neutral genetic diversity

**DOI:** 10.1002/ece3.4436

**Published:** 2018-08-25

**Authors:** Haruna Nakamura, Kosuke Teshima, Hidenori Tachida

**Affiliations:** ^1^ Graduate School of Systems Life Sciences Kyushu University Fukuoka Japan; ^2^ Department of Biology Faculty of Science Kyushu University Fukuoka Japan

**Keywords:** cyclic size changes, demographic history, DNA polymorphism, population expansion, population shrinkage, Tajima's *D*

## Abstract

Recurrent changes in population size are often observed in nature, influencing the efficiency of selection and consequently affecting organismal evolution. Thus, it is important to know whether such changes occurred in the past history of a focal population of evolutionary interests. Here, we focused on cyclic changes in population size and investigated the distributional properties of Tajima's *D* and its power to distinguish a cyclic change model compared with the standard neutral model, changing the frequency and magnitude of the cyclic change. With very low or very high frequencies of the cycle, the distribution of Tajima's *D* was similar to that in a constant size population, as demonstrated by previous theoretical works. Otherwise, its mean was negative or positive, and its variance was smaller or larger depending on the time of sampling. The detection rate of the cyclic change against the constancy in size by Tajima's *D* depended on the sample size, the number of loci, and the time of sampling in addition to the frequency and amplitude of the cycle. Using sequence data of several tens of loci, the detection rate was fairly high if the frequency was intermediate and the sampling was made when population size was large; otherwise, the detection rate was not high. We also found that cyclic change could be discriminated from simple expansion or shrinkage of a population by Tajima's *D* only if the frequency was in a limited range and the sampling was made when the population was large.

## 1 INTRODUCTION

Although simple models of population genetics often assume that population size is constant, the size of natural populations is thought to change recurrently through time. This notion seems intuitively obvious, but there are also many observations that support it. For example, Kendall, Prendergast, and Bjørnstad (1998) estimated that approximately 30% of animal populations exhibited statistically significant periodicity in size. In addition, fossil records have demonstrated that organismal populations shrank and expanded in the past due to environmental changes or other reasons (Bennett, 1997). Such changes would affect levels and patterns of genetic variation, evolution of genes, and consequently the processes of organismal evolution (Ohta, 1972). Therefore, it is important to know how changes in population size affect the evolution of genes and to infer the history of size change in natural populations.

By tradition, population genetics have addressed changes in population size using a notion of effective population size (see a review by Charlesworth, 2009). In particular, if population size changes through time, neutral genetic variation in the population can be computed by considering a surrogate population with its size being the harmonic mean of the past population sizes. Although we can compute a certain quantity, such as the average heterozygosity, using the effective size thus defined, the validity of the assumption of using a surrogate population with a constant size to understand the dynamics of genetic variation in a population with varying size is dependent on the timescale of the changes (Nordborg & Krone, 2002).

Indeed, Sjödin, Kaj, Krone, Lascoux, and Nordborg (2005) demonstrated that the coalescent process of neutral alleles in a model with stochastic changes in population size converges to that in a model with constant size as the rate of the size change approaches zero or infinity; otherwise, there is no corresponding model with constant size that results in the same genealogical process. In addition, using simulation, they computed the expectation of one of the neutrality statistics, Fu & Li's F* (Fu & Li, 1993), as a benchmark to measure deviation from the constant size model and investigated effects of the change in population size on the statistic. From the results, they suggested that the population behaves as a constant size population if the rate of size change with the time measured in units of the present population size is very low or high, that is, in general, smaller than 1/10 or larger than 10. The genealogy under the model with recurrent size changes was further analyzed by Erikkson, Mehling, Rafajlovic, and Sagitov (2010). They derived a general formula to compute the moments of the total branch length in the genealogy. Assuming a sinusoidally varying population size as a concrete example, they computed the mean and other moments up to the fourth of the total branch length and demonstrated how these moments in a population with varying size deviate from those in the constant size population as the frequency of size change varies.

Although these studies clarified the genealogical structure in populations with size change, there are still some gaps between their results and their application in the interpretation of real polymorphism data, especially in detecting past recurrent changes in population size. First, we can only observe sequence differences among sampled alleles and cannot directly estimate their genealogy. Therefore, we want to know properties of the statistics calculated from sequence data. Second, to detect past changes in population size from polymorphism data, we need to know the distributional property (the variance at a minimum) of the statistics. Sjödin et al. (2005) computed the mean of Fu & Li's F* but did not calculate the variance. At last, simple expansion or shrinkage of a population results in deviations of statistics from those in the population with a constant size. Therefore, we would like to know whether such simple changes in population size could be discriminated from recurrent changes in population size using some of the statistics of polymorphism data.

To narrow the gaps mentioned above, we consider a population with cyclic change in size and investigate the distributional properties of Tajima's *D* (Tajima, 1989a) and the possibilities of detecting recurrent changes in population size using polymorphism data at a few dozen loci. There are a few reasons why we choose a simple cyclic change model from a variety of possible models with recurrent size changes. First, a significant proportion of animal populations exhibit cyclic change in population size (Kendall et al., 1998). Second, some abiotic and biotic environmental changes are cyclic; for example, temperature and humidity change with a periodicity of approximately one hundred thousand years in glacial cycles (Bennett, 1997). In addition, host–parasite interaction often results in cyclic changes in population size (e.g., Stahl, Dwyer, Mauricio, Kreitman, & Bergelson, 1999). Depending on the generation time of the organism, the frequencies of those environmental changes measured in units of one generation vary among organisms. Third, although changes in population size are typically not exactly cyclic but involve some elements of stochasticity, we cannot employ the stochastic model used by Sjödin et al. (2005) because we want to consider cases where samples of alleles at multiple loci are taken from a population whose size at each past time point takes a specific value. If we employ their model, the variance of a statistic involves not only that from genetic drift but also that from the stochastic change in population size. At last, we can observe the effects of the rate of size change on genetic diversity most easily by employing cyclic change.

In this study, we investigate the following questions assuming a model with cyclic change in population size using simulation, changing the frequency and amplitude of the cycle. First, we investigate the effects of the frequencies and amplitude of the cycle and the timing of sampling on the distribution of Tajima's *D*. Second, we study the effects of the number of loci, the mutation rate, and the parameters of the cyclic change on the power of a test based on the mean of Tajima's *D* to discriminate the cyclic change model from the constant size model, which is hereafter referred to as the standard neutral model. We also investigate the power of a likelihood ratio test using a model‐based inference method for the demographic history developed by Excoffier, Dupanloup, Huerta‐Sánchez, Sousa, and Foll (2013) and compare the powers of the two tests. Third, because deviation of Tajima's *D* is also observed in models with simple population expansion or shrinkage (Tajima, 1989b), we compare the mean and variance of Tajima's *D* in the cyclic change model with those simple size change models. We find that the distribution of Tajima's *D* approaches that under the standard neutral model when the frequency of the cycle is very low or very high as noted by previous theoretical works (Erikkson et al., 2010; Sjödin et al., 2005), but the approach depends on the timing of sampling in addition to the frequencies and amplitude of the cycle. If the sampling is made when the population size is large, deviation of the cyclic change model with intermediate frequencies from the standard neutral model can be detected using data of several tens of loci, and discrimination from simple expansion may be possible under some condition. Otherwise, detection becomes more difficult, and the cyclic change is indistinguishable from simple shrinkage. Considering the importance of past population structure on the consequences of natural selection and ease of obtaining data at more than dozens of loci in nonmodel organisms (Lascoux & Petit, 2010), examining neutrality statistics, such as Tajima's *D*, to obtain knowledge of past population size in addition to levels of genetic diversity and differentiation will be helpful as a first step for understanding the evolution of a target species.

## 2 METHODS

We chose Tajima's *D* (Tajima, 1989a) as a statistic to detect recurrent changes in population size. We also examined distributional properties of various other statistics, such as Fu & Li's F* (Fu & Li, 1993) and Fay & Wu's *H* (Fay & Wu, 2000), for a small number of parameter sets that characterize recurrent changes, but Tajima's *D* generally exhibited the smallest variance when the population size was altered (data not shown). Tajima's *D* was originally developed to detect signals of selection, but it has been often used for inferring the demographic history (Ramos‐Onsins & Rozas, 2002). Tajima's *D* measures the difference between two unbiased estimators of the population mutation rate (*θ*), *k* and ${\hat{\theta }}_{W}$ (Tajima, 1989a), where *k* is the average number of pairwise nucleotide differences, and ${\hat{\theta }}_{W}$ is an unbiased estimator of *θ* (Watterson, 1975) calculated from the number of segregating sites, *S*. Tajima's *D* is defined by

$$D=\frac{k-{\hat{\theta }}_{W}}{\sqrt{e_{1}S-e_{2}S\left(S-1\right)}},$$

where *e*_1_ and *e*_2_ are calculated from the sample size *n*. Tajima's *D* tends to be negative in expanding populations and positive in shrinking populations (Tajima, 1989b).

We assumed the infinite site neutral Wright–Fisher model. We generated sample sequences under the standard neutral model, cyclic change model, population expansion model, and population shrinkage model for a panmictic population using the *ms* program (Hudson, 2002).

**Figure 1:**
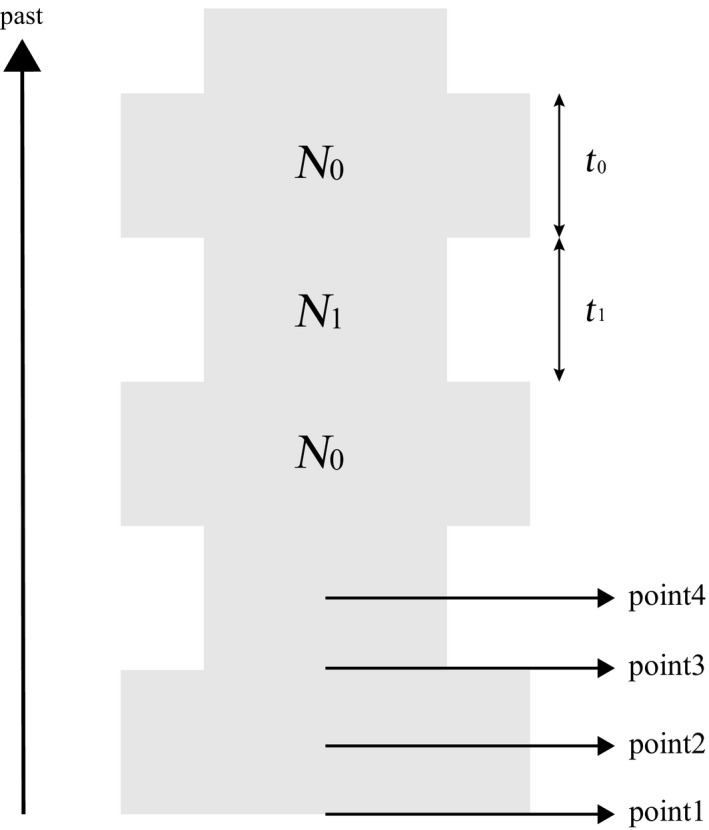
Cyclic change model. Population size cyclically changes between *N*_0_ and *N*_1_ over time. The sampling point, point *i*, is shifted by (*i*−1)/4 cycles from point 1. The size at sampling is *N*_0_ at point 1 and point 2 and *N*_1_ at point 3 and point 4. The length of the period when the size is *N*_0_ is the same as that when the size is *N*_1_, that is, *t*_0_ = *t*_1_

The cyclic change model used here is presented in Figure 1. Population size is *N*_0_ for *t*_0_ generations, suddenly changes to *N*_1_ (*N*_0_ > *N*_1_), and remains at that size for *t*_1_ generations. This is one cycle, and this process is repeated indefinitely. At a certain point in one of the cycles, *n* alleles are sampled. We set the mutation rate so that the expectations of *k* at the sampling point were the same when different models were compared.

In the simulation, we used *N*_0_/*N*_1_ = 5, 10, and 20 by varying the value of *N*_1_ but *N*_0_ = 100,000. The length (*t*_0_) of the period during which the size was *N*_0_ in a cycle was the same as that (*t*_1_) of the period during which the size was *N*_1_. We defined the frequency of cycle *i* as the number of cycles per *N*_0_ generations such that *t*_0_ =* t*_1_ = *N*_0_/(2*i*). The value of *i* examined ranged from 0.01 to 1,000. For example, when *N*_0_ is 100,000, *i *=* *1 indicates that the length of one cycle is 100,000 generations. We examined four sampling points, point 1 (at the end of the large phase), point 2 (at the midpoint in the large phase), point 3 (at the end of the small phase), and point 4 (at the midpoint in the small phase) as shown in Figure 1.

First, to assess effects of the frequency of the cycle (*i*) and the amplitude (*N*_0_/*N*_1_) of changes in population size and the timing (point *x*) of sampling on the distribution of Tajima's *D*, we generated 100,000 datasets of *n* sequences under the cyclic change model using *ms*. We first set E[*k*], the expectation of *k*, to 5.0 per locus at the sampling point. We computed the mutation rate for a specified E[*k*] at the sampling time using the formula (10) in Chakraborty (1977). We used a sample size of *n *=* *50. We computed the distribution, mean, and variance of Tajima's *D*.

Next, we investigated the power to discriminate the cyclic change model from the standard neutral model using the mean of Tajima's *D* when data from multiple loci were available. We followed the fixed *S* procedure of Hudson (1993) to test the generated datasets against the standard neutral model. The fixed *S* procedure simulates samples by fixing the number of segregating sites *S* instead of using the unknown parameter θ. This procedure seems reliable in cases without recombination (Wall & Hudson, 2001). We calculated the power in the following manner. (a) Generate a dataset of *n* samples at each of a specified number of loci assuming the cyclic change model. (b) Calculate the mean of Tajima's *D* across the loci. (c) Generate a dataset of *n* samples at each of the specified number of loci with the observed *S* assuming the standard neutral model. (d) Calculate the mean of Tajima's *D* across the loci. (e) Repeat (c) and (d) 1,000 times to obtain the null distribution of the mean of Tajima's *D*. (f) Examine whether the mean of Tajima's *D* under the cyclic change model is outside the 95% critical region. (g) Repeat (a)–(f) 1,000 times to calculate the power of Tajima's *D* for detecting cyclic change. The power depends on the number of loci, sample sizes, E[*k*] at the sampling time and the demographic parameters. We investigated the cases with the number of loci = 10, 20, 50 and 100, E[*k*] = 0.5, 1.0 and 5.0 and the frequency of the cycle *i *=* *0.05, 0.1, 0.5, 1, 5, 10, 50 and 100. For sample size *n*, we examined only the case of *n *=* *50 because Tajima's *D* is not sensitive to deviations if *n* is less than 30 (Tajima, 1989a). As sample size increases, the probability of detecting deviations from the standard neutral model increases (Simonsen, Churchill, & Aquadro, 1995; Sjödin et al., 2005). Therefore, we also expect that the power of Tajima's *D* to detect cyclic change increases with sample size.

To compare this simple method of using Tajima's *D* to detect cyclic change with more sophisticated model‐based methods for inference of the demographic history, we used the likelihood ratio test implemented in *fastsimcoal2* (Excoffier et al., 2013) and examined its power to detect cyclic change against the standard neutral model. Because it took a long time for each testing using *fastsimcoal2*, we repeated the test only 100 times to calculate its power with the number of loci set to 100.

At last, we compared the mean and variance of Tajima's *D* across 100,000 loci in the cyclic change model with those in simple population expansion and shrinkage models. Simulations under these models were performed using methodology similar to that used for the cyclic change model. In the population expansion (shrinkage) model, the size was *N*_1_ (*N*_0_) in the past but suddenly changed to *N*_0_ (*N*_1_) at *t* generations ago. In the simulation, we used *N*_0_/*N*_1_ from 1 to 1,000. The time until the change *t*/*N*_0_ (*t*/*N*_1_) was set to values from 0.00005 to 50 (from 0.005 to 50) in the population expansion (shrinkage) model.

In evaluating the power of the tests, we included monomorphic data obtained from the simulation; otherwise we excluded monomorphic data.

## 3 RESULTS

### 3.1 Effects of cyclic size changes on Tajima's *D*

**Figure 2:**
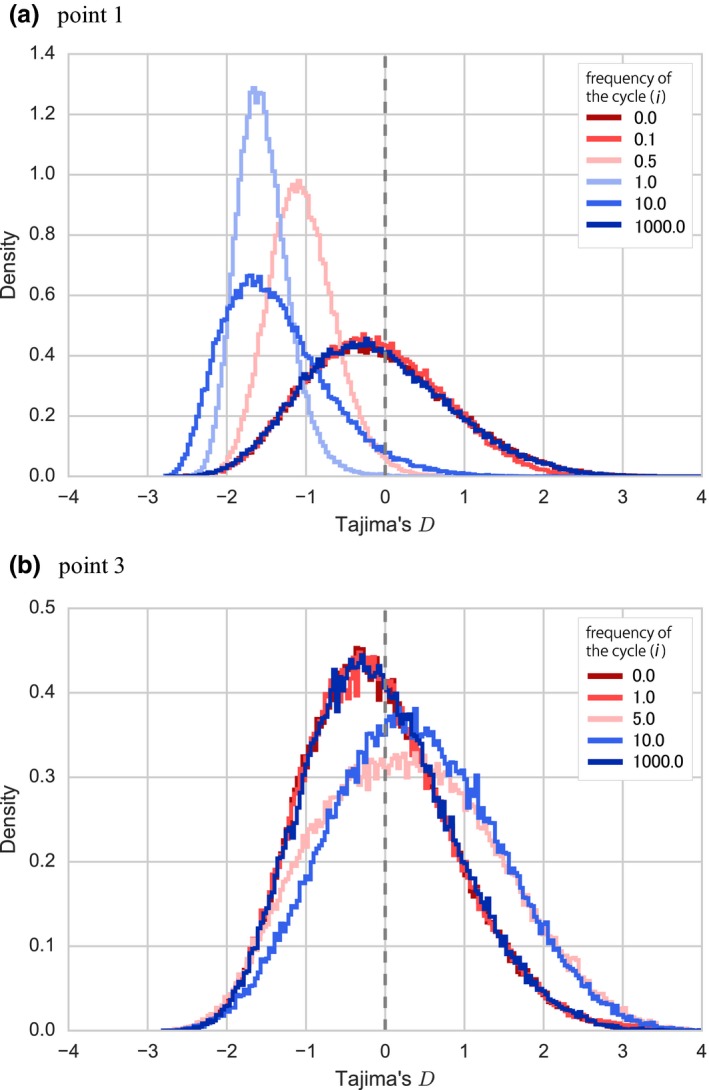
The distribution of Tajima's *D* in each sampling point. For each combination of a sampling point and frequency of the cycle, 100,000 datasets were generated with sample size *n *=* *50, amplitude *N*_0_/*N*_1_ = 20, and *E*[*k*] = 5.0. Monomorphic datasets were excluded. The upper‐right inset indicates the color used for the number of cycles per *N*_0_ generations. Red lines indicate the distributions under the standard neutral model

Figure 2 presents the change of the distribution of Tajima's *D* when the frequency of the cycle was changed. When sampling was made at point 1, the distribution first shifted toward negative as the frequency increased from zero and then reversed its direction as the frequency further increased, ultimately approaching that of the standard neutral model. When sampled at point 2, the distribution exhibited similar behavior (not shown). On the other hand, when sampling was made at point 3, it shifted toward positive and reversed its direction as the frequency increased, ultimately approaching that under the standard neutral model. A similar result was obtained when sampling was performed at point 4. Of note, the mean and the variance varied depending on the frequency in all cases.

**Figure 3:**
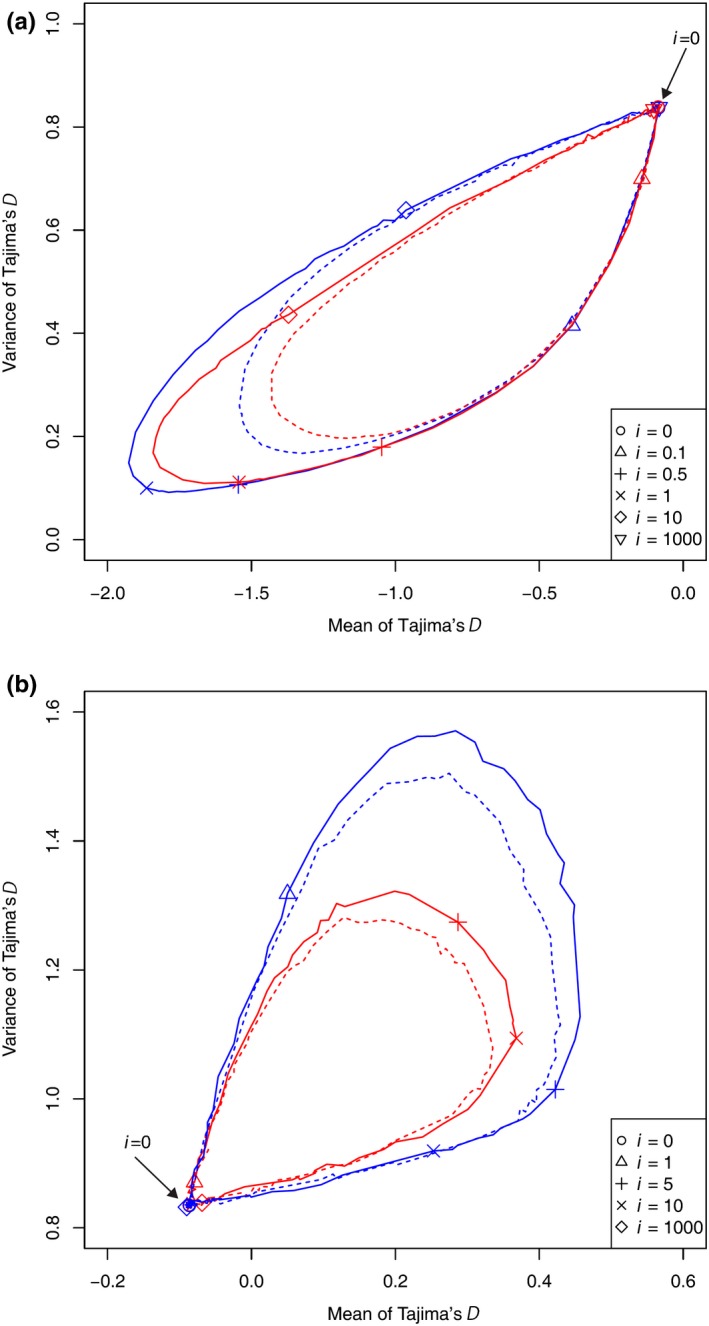
The mean and variance of Tajima's *D* depending on the frequency of the cycle. For each sampling point, 100,000 datasets were generated with sample size *n *=* *50, amplitude *N*_0_/*N*_1_ = 20 (solid line) and 10 (short dashed line), and E[*k*]* *= 5.0. Monomorphic datasets were excluded in the calculation. Symbols indicating some of the frequencies are explained in the lower‐right inset. (a) Red and blue lines represent sampling at point 1 and point 2, respectively. (b) Red and blue lines represent sampling at point 3 and point 4, respectively

To present the cases under different parameter values, we summarized the distributional property using the mean and variance and plotted these data in Figure 3 for various frequencies of the cycle and sampling points with the amplitude *N*_0_/*N*_1_ = 10 and 20. When sampling was performed at time point 1 or point 2 (when population size was *N*_0_), the mean and variance of Tajima's *D* exhibited a clockwise rotation (negative mean and smaller variance) as the frequency of the cycle increased from zero (Figure 3a). As the frequency further increased, the mean and variance of Tajima's *D* both increased and finally approached those under the standard neutral model. Of note, the same mean was observed at two different frequencies but the variances were different. When sampling was performed at time point 3 or point 4 (when population size was *N*_1_), a clockwise rotation was also observed, but the mean and variance both increased first and then decreased (Figure 3b). In all cases, if we decreased the amplitude, the oval became smaller (Figure 3).

### 3.2 Detection of cyclic change using data from a finite number of loci

**Figure 4:**
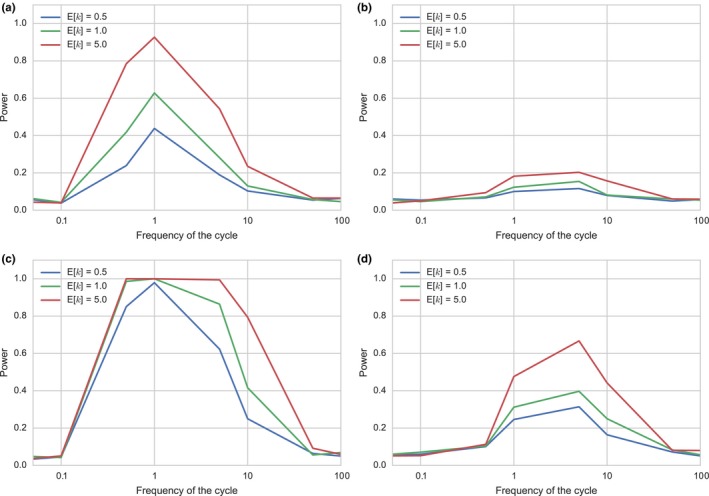
The effects of the number of loci and expected genetic diversity at sampling points on the power to detect cyclic change. For each sampling point, the power was calculated with *n *=* *50, *N*_0_/*N*_1_ = 5 and E[*k*] = 0.5, 1.0, and 5.0. (a) Sampling at point 1, number of loci = 10; (b) sampling at point 3, number of loci = 10; (c) sampling at point 1, number of loci = 50; (d) sampling at point 3, number of loci = 50

We next examined under what condition cyclic change was detected when tested against the standard neutral model using the mean of Tajima's *D* as a test statistic. Figure 4 presents the effects of the frequency of the cycle, sampling time point, the mutation rate and the number of loci on the power to detect cyclic change when the amplitude *N*_0_/*N*_1_ was 5. Cyclic change was detected when the frequency exhibited intermediate values, as noted by Sjödin et al. (2005). The power was high when the number of loci was large (Figure 4c,d), the mutation rate is high and sampling was performed at point 1 (or point 2; data not shown). If the number of loci was 10 (Figure 4a,b), the power was very low when sampling was performed at point 3. In contrast, the power was fairly high when sampling was performed at point 1. Thus, data of many loci are required to detect cyclic change if sampling is performed when the population size is small.

**Figure 5:**
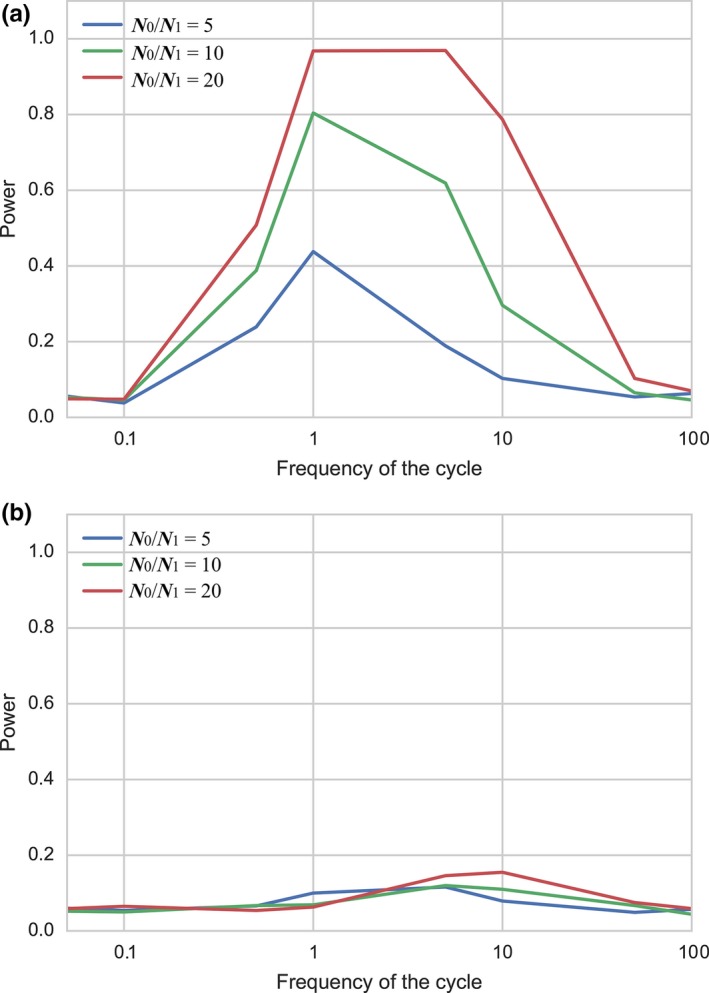
Effects of the magnitude and sampling point on the power to detect cyclic change. For each sampling point, the power was calculated with *n *=* *50, E[*k*] = 0.5, and the number of loci = 10. (a) Sampling at point 1, (b) sampling at point 3

The effect of changing the amplitude on the power was also investigated (Figure 5). The amplitude *N*_0_/*N*_1_ was changed from 5 to 20 with the number of loci set to 10. When *N*_0_/*N*_1_ = 20, the cyclic change was very likely to be detected if the frequency was between 1 and 10. Although the large amplitude greatly increased the power when sampling was made at point 1, it did not significantly affect the power when sampling was performed at point 3 and the probability of detection was almost nil when the number of loci examined was low (10).

**Figure 6:**
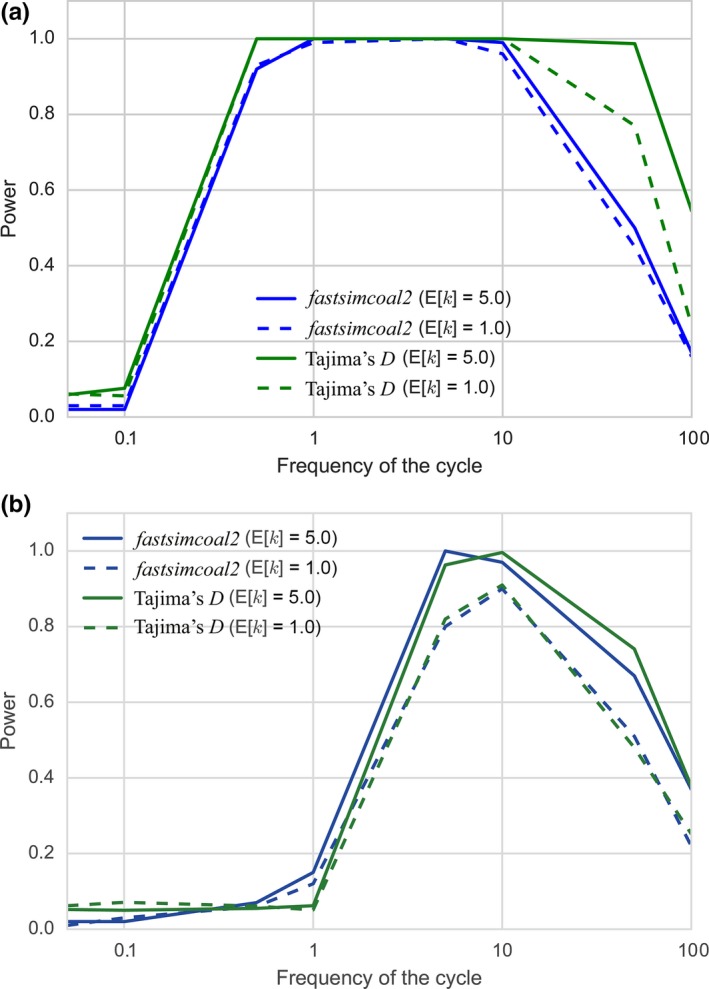
Comparison between powers of the tests using Tajima's *D* and *fastsimcoal2* changing E[*k*]. For each sampling point, the power was calculated with *n *=* *50, *N*_0_/*N*_1_ = 5, the number of loci = 100, and E[*k*] = 1.0 and 5.0. (a) Sampling at point 1; (b) sampling at point 3

**Figure 7:**
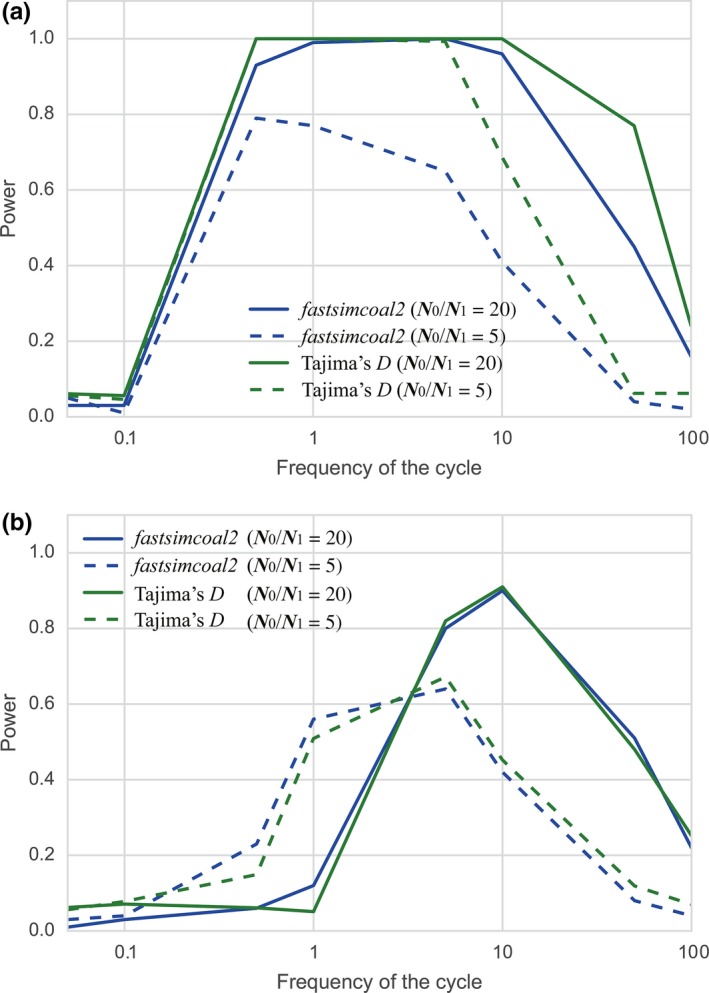
Comparison between powers of the tests using Tajima's *D* and *fastsimcoal2* changing amplitude. For each sampling point, the power was calculated with *n *=* *50, *N*_0_/*N*_1_ = 5 and 20, the number of loci = 100 and E[*k*] = 1.0. (a) Sampling at point 1; (b) sampling at point 3

The power of detecting the cyclic change against the standard neutral model using the likelihood ratio test implemented in *fastsimcoal2* was also investigated and the results together with those using Tajima's *D* are shown in Figure 6 (changing E[*k*]) and in Figure 7 (changing the amplitude). The power of the test based on Tajima's *D* was higher (sampling point 1) or comparable (sampling point 3) compared to that of the likelihood test. Moreover, the latter test took seventy times more computational time than the former test. Therefore, the test based on Tajima's *D*, although it does not provide estimates of parameters, is considered to be more efficient than the likelihood test to detect cyclic change against the standard neutral model.

### 3.3 Comparison between cyclic change and simple demographic change models

**Figure 8:**
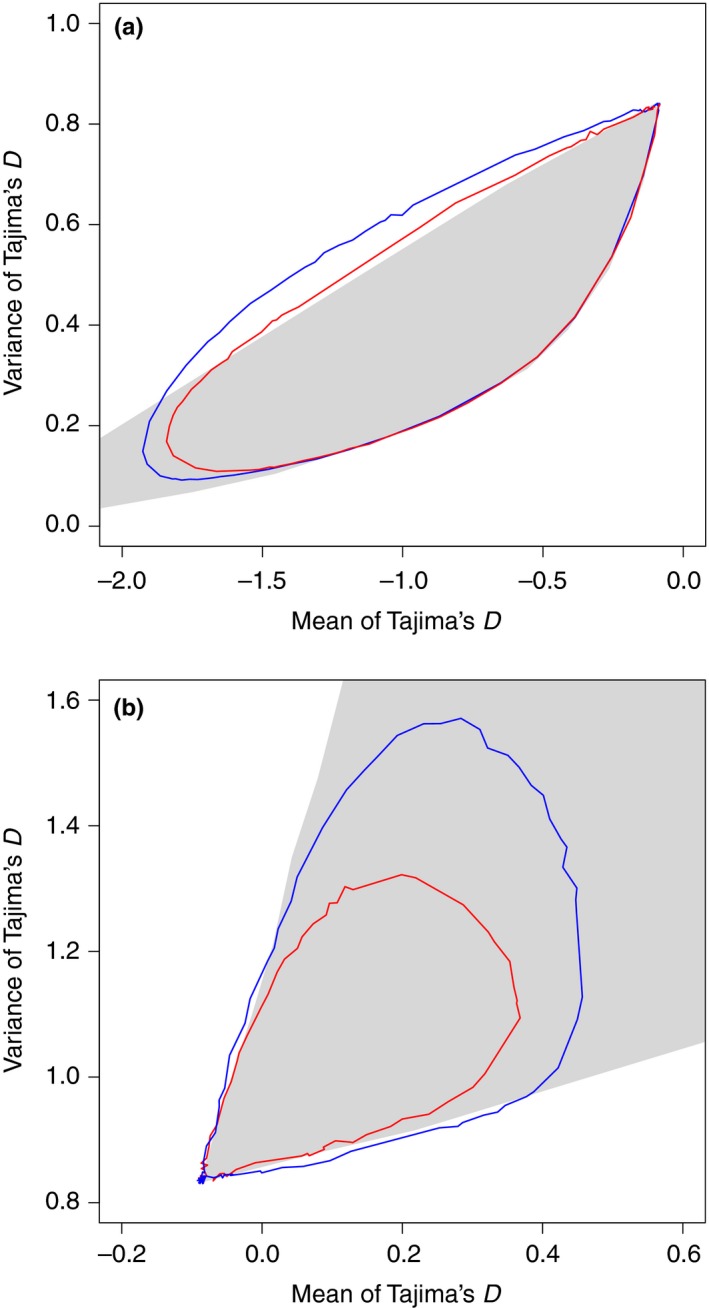
Comparison between cyclic change and simple demographic changes. (a) The mean and variance under the cyclic change model with *N*_0_/*N*_1_ = 20, and various frequencies are indicated by the red (sampling at point 1) and blue (sampling at point 2) lines. Those under the simple expansion model with various parameter values are confined to the gray‐colored region. Sample size *n *=* *50 and E[*k*] = 5.0. (b)The mean and variance under the cyclic change model with *N*_0_/*N*_1_ = 20 and various frequencies are presented using the red (sampling at point 3) and blue (sampling at point 4) lines, and those under the simple shrinkage model with various parameter values are confined to the gray‐colored region. Sample size *n *=* *50 and E[*k*] = 5.0

We compared the mean and variance of Tajima's *D* under the cyclic change model with those under the population expansion model (Figure 8a). The mean and variance of Tajima's *D* under the cyclic change model can exhibit values outside the region of values under the population expansion model. Under the cyclic change model with high frequencies, if sampling was performed at point 2 (but not at point 1), the variance exhibited a value larger than the upper limit of the population expansion model with the same mean of Tajima's *D*. Therefore, in this case, we can discriminate the cyclic change model from the population expansion model.

If sampling was performed when the population was small, the mean and variance of Tajima's *D* under the cyclic change model were within the region observed under the population shrinkage model (Figure 8b). Those values under the population shrinkage model could take any points in the region with an appropriate parameter set of *t* and *N*_0_/*N*_1_. Therefore, when sampling was performed at point 3 or 4, we cannot discriminate the cyclic change model from the population shrinkage model by simply estimating the mean and variance of Tajima's *D*.

## 4 DISCUSSION

If population genomic data with reliable genomic resources are available as in model organisms, such as human, *Drosophila* and *Arabidopsis* (Langley et al., 2012; The 1000 Genomes Project Consortium, 2012; The 1001 Genomes Consortium, 2016), we can use some of the model‐flexible methods (Ho & Shapiro, 2011; Liu & Fu, 2015) to infer recurrent changes in population size in the past. For many nonmodel organisms of evolutionary and/or ecological interests, only limited data of a dozen or up to a few hundred short gene segments are available (Lascoux & Petit, 2010). In such cases, these model‐flexible methods are not suitable for inferring recurrent changes in population size. One can use the Extended Bayesian Skyline Plot (Heled & Drummond, 2008), but the method consumes a large amount of computational time if the number of loci is dozens or larger. Instead, one may use model‐based methods (e.g., Beaumont, Zhang, & Balding, 2002; Excoffier et al., 2013), but it takes a fairly large amount of computational time to test cyclic changes of a population and the power of detection is not necessarily high as shown here. Indeed, it took 70 times more time to test cyclic changes using the likelihood ratio test implemented in *fastsimcoal2*, one of the most frequently used model‐based methods for inference of the demographic history, than using the test based on the mean of Tajima's *D* as shown here. Therefore, a simple method based on a neutrality statistics such as Tajima's *D* seems still useful for theoretically studying possibilities of detecting cyclic changes and also applying it to limited data of nonmodel organisms.

In the present study, assuming cyclic changes in population size, we investigated the distributional properties of Tajima's *D*. First, its power to detect the cyclic change depended on the frequency of the cycle. When the frequency was very low or very high, the distribution of Tajima's *D* was very similar to that of the standard neutral model with its current or harmonic mean effective size, respectively, as previously demonstrated analytically by Sjödin et al. (2005) and Erikkson et al. (2010). However, when the frequency was intermediate, the mean and the variance of the statistic deviated from those of the standard neutral model. The deviation of Tajima's *D* was observed when the frequency measured with time units of *N*_0_ generations was between 1/10 and 100, but the range of the frequency in which the deviation was observed depended on the timing of the sampling. Second, the power to detect cyclic change using Tajima's *D* depended on the frequency and amplitude of the cycle, the time point of sampling, and numbers of sampled alleles and loci. If we obtain 50 sequences at each of 50 loci, the detection rate of cyclic change with a frequency between 0.3 and 10 was high if the sampling was performed when the population was large. However, the detection rate was low if the sampling was performed when the population was small. At last, if sampling was performed when population was large, cyclic change with a large amplitude and higher frequencies might be discriminated from simple expansion by examining the mean and variance of Tajima's *D*. However, it was otherwise difficult to discriminate the cyclic change from simple size changes by exclusively examining Tajima's *D*. These observations, taking a typical case of E[*k*] = 1.0, are summarized in Table 1.

**Table 1:** Frequencies at which cyclic change can be detected^a^

*l* b	*N*_0_/*N*_1_	Sampling time	
		Point 1, 2	Point 3, 4
10	5	1.0	None
	10	0.5–5.0	None
	20	0.5–10.0	None
50	5	0.5–5.0	None
	10	0.5–10.0	5.0–10.0
	20	0.5–50.0	5.0–10.0
Discrimination^c^		Conditionally possible	No

^a^Frequencies of the cycle at which detection is possible with *p *>* *0.5 are shown. E[*k*] = 1.0 and *n *=* *50. ^b^Number of loci. ^c^Discrimination from simple size change models (point 1, 2, expansion; point 3, 4, shrinkage).

Erikkson et al. (2010) computed up to the fourth moments of the total branch lengths of the genealogy assuming cyclic change in population size. It is interesting that Figure 4 in their paper demonstrates that the second and higher moments converge to those of the standard neutral model with the harmonic mean population size when the frequency of the size change is greater than 1. In contrast, the variance of Tajima's *D* differed from that of the standard neutral model even when the frequency is 10 or greater. Tajima's *D* is a function of the shape of the genealogy characterized by quantities, such as the ratio of the lengths of the internal and external branches. This finding demonstrates that the distributional properties of statistics or random variables associated with the genealogy in a population with size change differ from one another, and analyses of various statistics directly related to the data are necessary.

In our analyses of cyclic change in population size, we made several simplifying assumptions. Here, we discuss a few of them briefly. First, we investigated effects of the cyclic change model with only equal lengths of the periods of large and small population sizes (*t*_0_ = *t*_1_), but this is not always the case. For example, one glacial cycle consists of a long period of a cool and dry climate and a short period of a warm and humid climate (Bennett, 1997). If the focal organism is adapted to the warmer period, the time when the population size is large becomes shorter (*t*_0_ < *t*_1_). Therefore, we need to consider the effects of unequal lengths of the periods. We can predict consequences of the inequality when the frequency of the cycle is low or high. If the size change is slow, Tajima's *D* is strongly affected by the time of the most recent change in population size. Thus, its behavior when *t*_0_ *≠ t*_1_ would be similar to that of the equal length case (*t*_0_ = *t*_1_) with the same length of time to the most recent size change. Hence, detection of cyclic change becomes possible from a lower frequency if the size at sampling persists shorter in a cycle and vice versa. On the other hand, when the frequency of the cyclic change is high, multiple cycles occur before the time of the MRCA (TMRCA). The convergence to the standard neutral model as the frequency increases results from the averaging over the cycles. Given that coalescences occur mostly when the population size is small, the number of cycles before the TMRCA increases as *t*_1_ becomes shorter in one cycle. Therefore, the convergence occurs at a lower frequency when *t*_0_ > *t*_1_ compared with the equal case and at a higher frequency when *t*_0_ < *t*_1_.

Second, we assumed that sudden changes in population size although natural populations would rarely grow 5 or 10 times larger in one generation. We made this assumption given that gradual growth in the transitional stage, which seems more realistic, increases the number of parameters. If the growth rate is high such that the transitional period is considerably shorter than the length of one cycle, differences between the two models would be negligible. Otherwise, the result will change. In a study investigating the power of various neutrality statistics in detecting simple population growth, Ramos‐Onsins and Rozas (2002) reported that the power to detect the growth is increased in the sudden growth model compared with the logistic population growth model. Although these one‐time change models may differ from our recurrent change model in various aspects in terms of their effects on the genealogy, this may also be the case for our model. First, effects of the amplitude of the cycle measured by the ratio of the maximum and minimum population sizes on the genealogy would be smaller in the gradual change model compared with the sudden change model because the ratio of the rates of coalescences in the two periods is smaller in the former if the amplitudes are the same. Regarding the effects of the frequency of size change on detection, we first assume for simplicity that coalescences occur only when the population is small and the frequencies in the gradual and sudden change models are the same. When the frequency is high, the power to detect recurrent changes would be reduced in the gradual change model compared with the sudden growth model because the number of coalescences in one cycle is smaller in the gradual change model. Thus, the number of cycles until the TMRCA increases. However, when the frequency is low, although the power under the sudden change model is increased compared with the gradual change model if sampling is performed when the population is large (Ramos‐Onsins & Rozas, 2002), it is lower if sampling is performed when the population is small because the time to the MRCA is reduced compared with the gradual change model with the same frequency and amplitude.

Third, we assumed no intragenic recombination in the analysis but recombination occurs especially when longer sequences, which reflect increased E[*k*] in our case, are used in analyses of natural populations. Given that the effects of introducing intragenic recombination are similar to those of increasing the number of loci, we would generally be able to detect deviation from the standard neutral model in wider ranges of the frequency and amplitude of the cycle (see, e.g., table 6 of Rozas, Segarra, Ribó, & Aguadé, 1999). However, effects of recombination are not restricted to averaging of genealogies along the sequence. As shown by Schaper, Eriksson, Rafajlovic, Sagitov, and Mehlig (2012), the covariance between the TMRCA of two gene segments does not converge to that of the standard neutral model with an effective size of the harmonic mean as the frequency of the cycle increases if the recombination rate is comparable to the rate of size change. Although the means of the neutrality statistics derived from the frequency spectrum, such as Tajima's *D*, do not change, their variance may be affected. Therefore, we need to investigate the effects of intragenic recombination under recurrent changes in population size in future studies.

Demographic changes in populations affect the consequences of natural selection, often significantly (Brandvain & Wright, 2016). For example, nearly neutral mutations (Ohta, 1992) behave like neutral alleles in small populations but as selected alleles in large populations, and recurrent changes in population size result in irregularities in the molecular evolution of such mutations (Cutler, 2000). In addition, the total number of advantageous mutations appearing in one generation is small in small populations but large in large populations, thus facilitating rapid adaptation in large populations. These considerations lead us to conclude that the evolutionary paths of populations with recurrent changes in size would significantly differ from those with constant size or with one‐time size change. Therefore, it is important to know whether recurrent size changes have occurred in the populations of the target organism by examining means and variances of neutrality statistics at neutrally evolving loci.

However, it is generally difficult to determine whether the loci at which the data are collected have been affected by selection or not. Of late, Ewing and Jensen (2016) pointed out that inferences on the demographic history would be strongly biased by intermediate levels of background selection. Background selection poses a serious problem in inferring the demographic history especially when population size changes because a wide range of fitness effects become intermediate sometime in the past. One way to avoid this problem may be to use RAD (restriction site‐associated DNA) sequencing (Baird et al., 2008) or related methods, whose target sites are mostly in noncoding regions and possibly neutrally evolving. The simple method using the mean of Tajima's *D* investigated here may be especially useful for data obtained by methods such as RAD sequencing because number of samples at each locus is variable and the frequency spectrum required, for example, for *fastsimcoal2,* is sometimes difficult to obtain.

Given the importance of knowing the past demographic history but recognizing the difficulty in its inference, we recommend that a preliminary analysis for data obtained by a method such as RAD sequencing using the mean of Tajima's *D* to detect deviation from the standard neutral model is carried out. This knowledge will help planning further studies on the target species using more data in terms of number of individuals and read depths of the sequencing, which may aim to estimate population parameters using model‐based methods or evaluate effects of selection on some or all part of the genome.

## CONFLICT OF INTEREST

None declared.

## AUTHOR CONTRIBUTIONS

H.N. and H.T. designed the research. H.N. and K.T. wrote the python codes for simulation and analyses. H.N. analyzed the data. H.N. and H.T. wrote the manuscript.

## DATA ACCESSIBILITY

All programs used in this study are available on the Dryad repository, DOI: https://doi.org/10.5061/dryad.364cf36.
